# Integrated Analysis Reveals Critical Ferroptosis Regulators and FTL Contribute to Cancer Progression in Hepatocellular Carcinoma

**DOI:** 10.3389/fgene.2022.897683

**Published:** 2022-05-16

**Authors:** Shaoying Ke, Congren Wang, Zijian Su, Shaoze Lin, Gongle Wu

**Affiliations:** Hepatological Surgery Department, First Hospital of Quanzhou Affiliated to Fujian Medical University, Quanzhou, China

**Keywords:** ferroptosis, ferritin light chain, hepatocellular carcinoma, progression, prognosis

## Abstract

**Background:** The carcinogenesis and prognosis of hepatocellular carcinoma (HCC) involve complex molecular mechanisms, and ferroptosis is related to the development and therapeutic efficacy of HCC, but the specific mechanism and prognostic role of ferroptosis-related genes in HCC have not been elucidated.

**Methods:** Differentially expressed gene analysis, Cox regression, and unsupervised consensus clustering were applied to identify crucial ferroptosis regulators and establish ferroptosis-related subtypes in HCC. Random forest analysis and survival analysis were adopted to confirm FTL as the hub prognostic and diagnostic ferroptosis regulator in HCC.

**Results:** The ferroptosis-related subtypes based on the crucial prognostic ferroptosis regulators showed that patients in fescluster A had a higher survival probability (*p* < 0.001) and better clinical characteristics than patients in fescluster B in the TCGA-LIHC cohort. Patients with a high tumor mutation burden (TMB) in fescluster B presented a significantly poorer prognosis. FTL was the core ferroptosis regulator, and its low expression revealed a significant survival advantage compared with its high expression (*p* = 0.03). The expression and predictive value of FTL were both closely related to the clinical features (*p* < 0.05). Expression of FTL accurately distinguished HCC from normal tissues in the TCGA-LIHC cohort, ICGC cohort, and GSE14520 dataset. In addition, higher infiltrating fractions of immune cells, such as activated CD8^+^ T cells and Gamma delta T cells, mainly enriched immune-related signaling pathways, including the IL2-STAT3 signaling pathway and interferon-gamma response signaling pathway, and higher expression of immune checkpoints, including PDCD1, CTLA4, TIGIT, and CD83, were presented in patients with high FTL expression (*p* < 0.05). Patients with high FTL were more sensitive to some targeted drugs, such as cisplatin, dasatinib, and sorafenib, than those with low FTL (*p* < 0.05). A nomogram based on FTL accurately predicted the prognosis of HCC. Further knockdown of FTL was determined to significantly inhibit cell proliferation and migration in HCC.

**Conclusion:** Our study validated ferroptosis-related subtypes and FTL with effective prognostic value in HCC and was beneficial for identifying candidates suitable for targeted drug therapy and immunotherapy, thereby offering further insight into individual treatment strategies to improve disease outcomes in HCC patients.

## Introduction

Hepatocellular carcinoma (HCC) is the most common type of primary liver cancer, which is the fifth most prevalent malignancy and the second leading cause of cancer-related death globally (Ferlay et al., 2015; Torre et al., 2015). HCC patients reveal various clinical symptoms, including weight loss, hepatalgia, diarrhea, obstructive jaundice, and ascites (Sun and Sarna, 2008; Chen et al., 2014). However, early HCC lacks classic clinical features, and once the signs and symptoms of liver cancer start to appear, most HCC cases are locally advanced and/or distant metastatic, which results in difficult therapy and poor prognosis (Gong and Qin, 2016; Ayuso et al., 2018).

Surgery, including liver transplantation (LT) and liver resection (LR), serves as an effective treatment for HCC. However, LT has strict selection criteria for patients, usually using the Milan standard (solitary tumor ≤5 cm and up to three nodules ≤3 cm) (Zarrinpar and Busuttil, 2013; Jadlowiec and Taner, 2016). LT is the most efficient therapeutic method, but the donor shortage greatly limits its applicability (Forner et al., 2018). Surgical resection is the most common therapy for HCC, but approximately 70% of cases experience a relapse within 5 years after surgical treatment (Hany et al., 2018). Chemotherapy is one of the most important treatment modalities for advanced HCC (Ikeda et al., 2018); however, unsolved issues remain, including drug resistance and metastasis to other organs. Therefore, it is crucial to identify molecular biomarkers that can be used for early diagnosis and prognosis prediction.

Ferroptosis is an iron-dependent form of nonapoptotic programmed cell death driven by disruption of the intracellular balance of glutathione peroxidase 4 (GPX4) degradation of lipid peroxides. In recent years, triggering ferroptosis in cancer has been found to be beneficial to cancer treatment, especially the effectiveness of drug-resistant cancer (Hassannia et al., 2019; Tang et al., 2022). Due to the high metabolic signature of cancer cells, they often show an increased requirement for iron, and their characteristic of “iron addiction” increases the likelihood that they trigger ferroptosis. There is limited effective drug therapy for HCC. Sorafenib is the only drug used for advanced HCC, but it is often unable to be further treated due to drug resistance. Current studies suggest that the negative regulator of ferroptosis, metallothionein-1g (MT-1G) (Sun et al., 2016a), the activation of nuclear factor erythroid 2-related factor 2 (NRF2) (Sun et al., 2016b), and the transcription factor yes-associated protein/transcriptional coactivator with PDZ-binding motif (YAP/TAZ) all inhibit ferroptosis (Gao et al., 2021), which may be the main mechanism of drug resistance in the treatment of HCC by sorafenib (Nie et al., 2018). The NRF2 inhibitor (alkaloid trigonelline) and the negative status of retinoblastoma (Rb) protein both enhance the sensitivity of ferroptosis, which might assist the effectiveness of sorafenib for HCC treatment (Arlt et al., 2013; Louandre et al., 2015). In addition, ferroptosis has a strong relationship with metabolism. Low-density lipoprotein-docosahexaenoic acid (LDL-DHA) induces ferroptosis in HCC by regulating lipid metabolism, and regulation of lactic acid mediated by hydroxycarboxylic acid receptor 1/monocarboxylate transporter 1 (HCAR1/MCT1) also affects ferroptosis (Ou et al., 2017; Zhao et al., 2020). The rapid development of biological information technology has helped us use computers to efficiently assist in the diagnosis and treatment of diseases. Most of the ferroptosis-related genes have been found to be closely related to the differentially expressed genes in HCC, and the ferroptosis and iron metabolism characteristic models are conducive to the diagnosis and prognosis prediction of HCC, as well as guiding the immunity or targeted therapy of HCC patients (Liu et al., 2020a; Liang et al., 2020; Tang et al., 2020). These studies are beneficial to the treatment of advanced HCC by ferroptosis of HCC. However, the mechanism of ferroptosis in HCC remains unclear.

Ferritin is an iron storage protein that participates in iron metabolism. There are two subunits of ferritin heavy chain (FTH) and ferritin light chain (FTL) in mammals, as well as the mitochondrial subunit form (FtMt), which exists only in mitochondria. FTL is composed of 174 amino acids with a molecular weight of 19 kDa, and its structure is more stable than that of FTH. Different FTH/FTL ratios have different functions. Iron storage organs such as the liver or spleen mainly contain FTL, while FTH is mainly related to antioxidant activity. As the main subunit of ferritin, FTL directly affects iron homeostasis (Arber et al., 2016). Current studies have found that FTL may be regulated by the DNA damage response of serine/threonine kinase ATM, hypoxia, atractylodin, and other factors, resulting in ferroptosis (Liu et al., 2020b; Chen et al., 2020; He et al., 2021). FTL may also be involved in the development of tumors, such as the proliferation rate of HeLa cells and glioblastoma multiforme (GBM) cells (Wu et al., 2016) and the drug resistance process of breast cancer (Cozzi et al., 2004). Circulating transcription of FTL is significantly upregulated in samples from HCC patients and maybe a new target for the diagnosis and treatment of HCC (Wang et al., 2009; Sayeed et al., 2020). The decrease in FTL protein is associated with ferroptosis in HCC cells (He et al., 2021), but its role and mechanism remain unknown.

In this study, we performed an integrative analysis of the molecular mechanism and prognostic role of 239 ferroptosis-related genes in HCC. Based on the prognostic ferroptosis regulators, consistent ferroptosis-related clusters were constructed. FTL served as a critical ferroptosis regulator by random forest analysis. Then, we explored the independent prognostic and diagnostic role of FTL and assessed the association of FTL with immune infiltration and immune checkpoints in HCC patients. In addition, we knocked down FTL in HCC cells to measure the oncogenic effect of FTL in HCC. These findings may help to explore the predictive role of FTL in the prognosis, diagnosis, therapeutic treatment, and oncogenesis of HCC patients.

## Materials and Methods

### Acquisition of Ferroptosis-Related Genes

The genes related to ferroptosis were downloaded from a previously published public data center (www.zhounan.org/ferrdb), and noncoding genes were removed from the dataset. The website also divided ferroptosis-related genes into three groups as follows: suppressors, drivers, and markers. We further analyzed the genes closely related to this research.

### Identification of Differentially Expressed Genes

We obtained the mRNA-sequencing data and clinical information of HCC patients from the liver hepatocellular carcinoma (LIHC) cohort in The Cancer Genome Atlas (TCGA) database (https://www.cancer.gov/), which included 19,676 annotated mRNA sequences and other clinical data from 370 tumor tissue samples and 50 normal tissue samples. After matching mRNA sequences with ferroptosis-related genes, the differentially expressed genes (DEGs) were selected by limma, an R package, with numerical conditions log2-fold change (FC)  >  1 and an adjusted *p*-value < 0.05.

### Identification of Molecular Subgroups by Consistent Clustering

We used the ConsensusClusterPlus package in R software for consistent clustering. The Euclidean squared distance metric and the K-means clustering algorithm were used to classify the TCGA-LIHC cohort into k clusters, with k = 2 to k = 9. The optimal number of clusters was determined by the consistent cumulative distribution function (CDF) graph and the delta region graph (Wilkerson and Hayes, 2010).

### Construction and Validation of a Predictive Nomogram

To predict the 1-, 3-, and 5-year survival probability of HCC patients, we combined all independent prognostic factors to construct a nomogram. Calibration curves were generated to assess the consistency between predicted survival rates and actual survival rates.

### Survival Analysis

The Kaplan-Meier (K-M) curve was a visualized tool for comparing the overall survival (OS) and progression-free survival (PFS) in different ferroptosis molecular subgroups, with log-rank tests to compare the curves. The receiver operating characteristic (ROC) curve, which was built by the R package “survivalROC,” was used to evaluate model prognostic performance by calculating the area under the ROC curve (AUC).

### Cell Culture

Human hepatocellular carcinoma cell lines, including SK-HEP1 and HCC-LM3, were obtained from the American Type Culture Collection (ATCC) (Manassas, VA, United States). The cells were cultured in DMEM (containing 10% fetal bovine serum and 100 U/ml penicillin–streptomycin) and placed in a 37°C, 5% CO_2_ incubator.

### Cell Transfection

We transfected FTL shRNAs into SK-HEP1 and HCC-LM3 cells through Lipofectamine 2000 (Invitrogen, CA, United States), which was synthesized by GeneChem (Shanghai, China). After cultivation in basic DMEM, the cells were cultured in DMEM supplemented with FBS and penicillin–streptomycin.

### Western Blotting

The total protein of the two liver cancer cell lines after FTL shRNA transfection was extracted using RIPA lysis buffer (Invitrogen) containing PMSF (Bio–Rad, Shanghai, China), and the protein concentration was determined and quantified. The total protein was treated with 10% sodium dodecyl sulfate–polyacrylamide gel electrophoresis (SDS–PAGE) and transferred to a polyvinylidene fluoride membrane (PVDF) (Invitrogen, Carlsbad, United States). After transfer, the membranes were blocked at room temperature for 2 h, then the primary antibody was added and incubated at 4°C overnight, and the secondary antibody was added and incubated at room temperature for 2 h. Finally, the iBright FL1500 intelligent imaging system (Invitrogen, Carlsbad, United States) was used to analyze the absorbance of the protein bands and calculate the relative protein expression level. The antibodies used in the study are listed in the Supplementary Material.

### Cell Proliferation Assay

SK-HEP1 and HCC-LM3 cells were digested and counted, seeded in 96-well plates (3,000 cells/plate in 200 µl DMEM), and cultured in a 37°C, 5% CO_2_ incubator. After 0, 24, 48, and 72 h, we washed the culture medium off the cells to be tested, added CCK8 solution according to the instructions, and continued to culture the cells for 2 h. The absorbance value (OD) of each group was measured at 450 nm on a microplate reader and recorded for statistical analysis. We also used the 5-ethynyl-2′-deoxyuridine (EdU) reagent (Ruibo, Guangzhou, China) and a viability/cytotoxicity kit (Invitrogen, Carlsbad, United States) according to the manufacturer’s protocol.

### Immunofluorescence

SK-HEP1 and HCC-LM3 cells were trypsinized for 24 h, rinsed with PBS three times (3 min/time), fixed with 4% paraformaldehyde for 15 min at room temperature, and then stabilized in 0.2% Triton for 10 min to rupture the cell membrane. Nonspecific antigen-binding sites were blocked with 2% BSA for 30 min, and then the cells were incubated with anti-PCNA overnight at 4°C. After washing, the cells were incubated with the anti-rabbit antibody for 60 min, and the nuclei were stained with DAPI for 2 min and then washed with PBS. Finally, a fluorescence microscope was used to observe and photograph the cells, and the expression levels of PCNA were detected.

### Statistical Analysis

The *t*-test was used for the measurement data, the χ^2^ test was used for the enumeration data, and the Kaplan-Meier method and log-rank test were used for the survival analysis. For all statistical calculations, the final results were determined to be statistically significant at *p* < 0.05.

## Results

### Identification of Prognostic Ferroptosis Regulators in HCC

**FIGURE 1:**
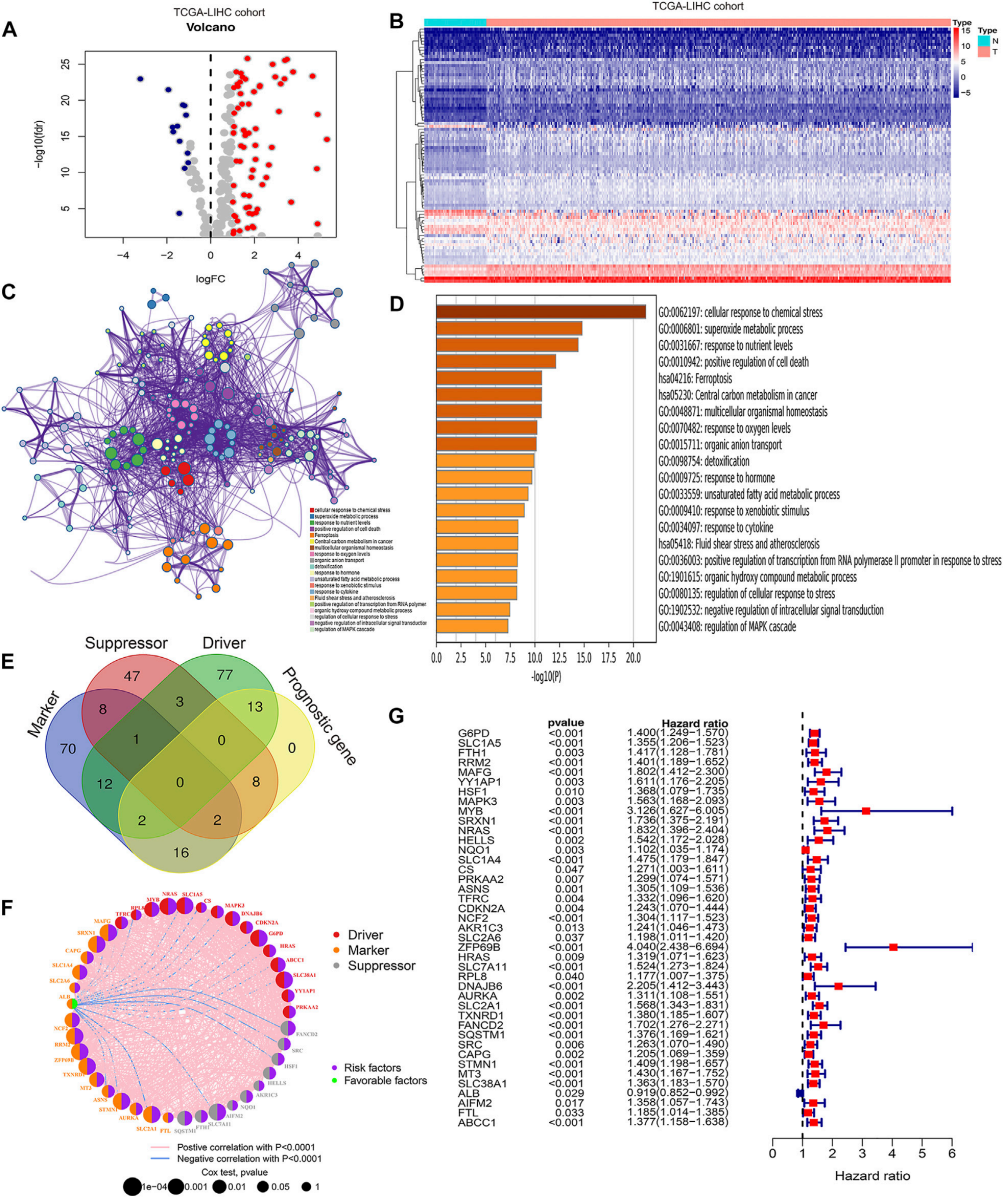
Identification of prognostic related ferroptosis regulators in HCC. **(A)** Volcano plot showing the expression characteristics of differentially expressed genes (DEGs) between tumor tissues and normal tissues in the TCGA-LIHC cohort. **(B)** Heatmap showing DEGs associated with ferroptosis in the TCGA-LIHC cohort. **(C)** Protein–protein network indicating the top 20 regulatory signaling pathways of DEGs via KEGG and GO functional analyses. **(D)** Bar plots showing the top 20 regulatory signaling pathways of DEGs via KEGG and GO functional analyses. **(E)** Venn diagram showing the function of ferroptosis-related genes. **(F)** Protein–protein network indicating the gene function, interaction, and prognostic role of DEGs. **(G)** Forest plot showing the prognostic value of DEGs using univariate Cox analysis.

To identify prognostic ferroptosis regulators in HCC, we first conducted differential gene expression analysis between tumor tissues and normal tissues in the TCGA-LIHC cohort, and the expression characteristics of the DEGs are shown in Figures 1A,B and Supplementary Table S1. Then, we performed Kyoto Encyclopedia of Genes and Genomes (KEGG) analysis and Gene Ontology (GO) analysis to explore the signaling pathways, biological processes, cellular components, and molecular functions of the DEGs. We confirmed “ferroptosis” as a mainly enriched signaling pathway (Figures 1C,D) and obtained a total of 239 ferroptosis-related DEGs that were enriched in the ferroptosis pathway. Subsequently, we drew a Venn diagram to identify the role of ferroptosis-related DEGs as markers, suppressors, and drivers in ferroptosis (Figure 1E). The gene function, interaction, and prognostic role among the ferroptosis-related DEGs are shown in Figure 1F. Using univariate Cox regression, we finally identified 41 prognostic ferroptosis regulators from markers, suppressors, and drivers of ferroptosis (Figure 1G).

### Establishment of Ferroptosis-Related Subtypes in HCC and Clinical Validation

**FIGURE 2:**
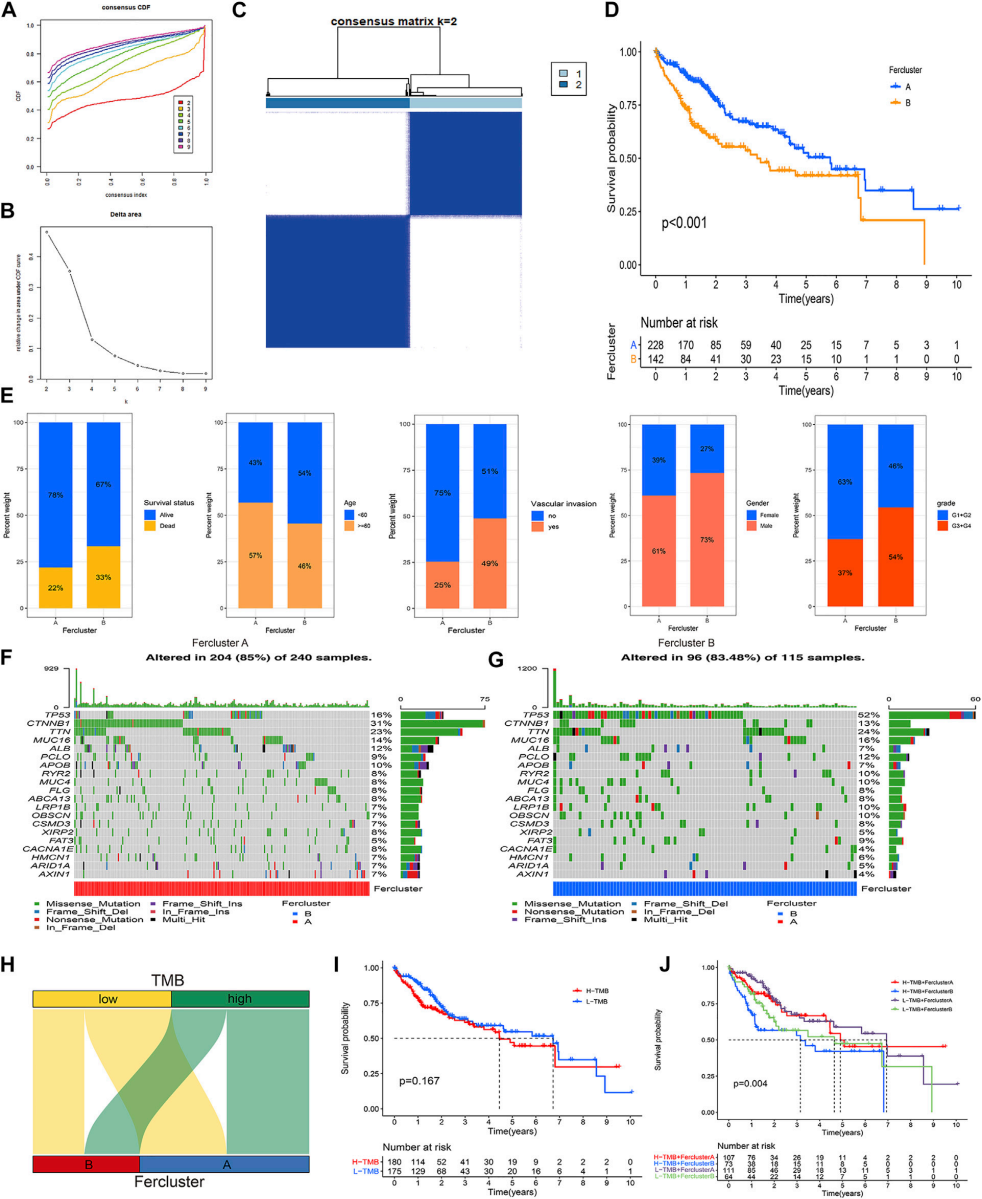
Establishment and clinical assessment of ferroptosis-related subtypes in HCC. **(A)** Consensus cumulative distribution function (CDF) curves with k from 2 to 9. **(B)** Delta area plot showing the relative change in area under the CDF curves between k and k-1 (k = 2–9). **(C)** The result of consensus clustering (k value = 2). **(D)** K-M curve showing the difference in survival probability between fescluster A and fescluster B. **(E)** Characteristics of clinical features in fescluster A and fescluster B. **(F–G)** Characteristics of TMB in fescluster A and fescluster B. **(H)** Venn diagram showing the correlation between TMB and fesclusters. **(I)** K-M curve showing the difference in survival probability between low and high TMB. **(J)** K-M curve showing the difference in survival probability among fescluster A with low TMB, fescluster A with high TMB, fescluster B with low TMB, and fescluster B with high TMB.

Unsupervised consensus clustering based on the expression characteristics of the 41 prognostic ferroptosis regulators was performed to establish ferroptosis-related subtypes in HCC. The results revealed that the optimal number of clusters was two (k value = 2) (Figures 2A–C). Hence, the patients in the TCGA-LIHC cohort were divided into two clusters, namely, fescluster A and fescluster B. The survival analysis indicated that compared with patients in fescluster B, patients in fescluster A showed a significant survival advantage (*p* < 0.001) (Figure 2D). We further explored the differences in clinical features between the two clusters, and the results revealed that patients in fescluster B had a higher proportion of death, age <60 years old, vascular invasion occurrence, and pathological grades G3/G4 than patients in fescluster A, and the patients in both fescluster A and fescluster B were predominantly male (Figure 2E). The results indicated that the clinical features of patients in fescluster B were worse than those in fescluster A. In recent years, tumor mutation burden (TMB), which represents the characteristics of the total number of somatic coding mutations in tumors, has been increasingly shown to have predictive value in tumor prognosis as a potential biomarker for non-small-cell lung cancer, liver cancer, and other cancer types (Rizvi et al., 2015; Tang et al., 2021). Based on this, we then confirmed the characteristics of TMB in fescluster A and fescluster B (Figures 2F,G) and explored the correlation between TMB and fesclusters (Figure 2H). The survival analysis shows that there was no statistically significant difference in survival probability between the high TMB group and the low TMB group (*p* > 0.05) (Figure 2I). We further divided the patients into fescluster A with low TMB, fescluster A with high TMB, fescluster B with low TMB, and fescluster B with high TMB, and survival analysis indicated that patients in fescluster A with low TMB showed a significant survival advantage compared with the other groups, while patients in fescluster B with high TMB had the worst prognosis (Figure 2J). These results revealed that the established ferroptosis-related subtypes in HCC showed effective prognostic predictive performance in HCC and were closely associated with the clinical characteristics of patients.

### FTL Is a Critical Ferroptosis Regulator Associated With Prognosis and Clinical Features in HCC

**FIGURE 3:**
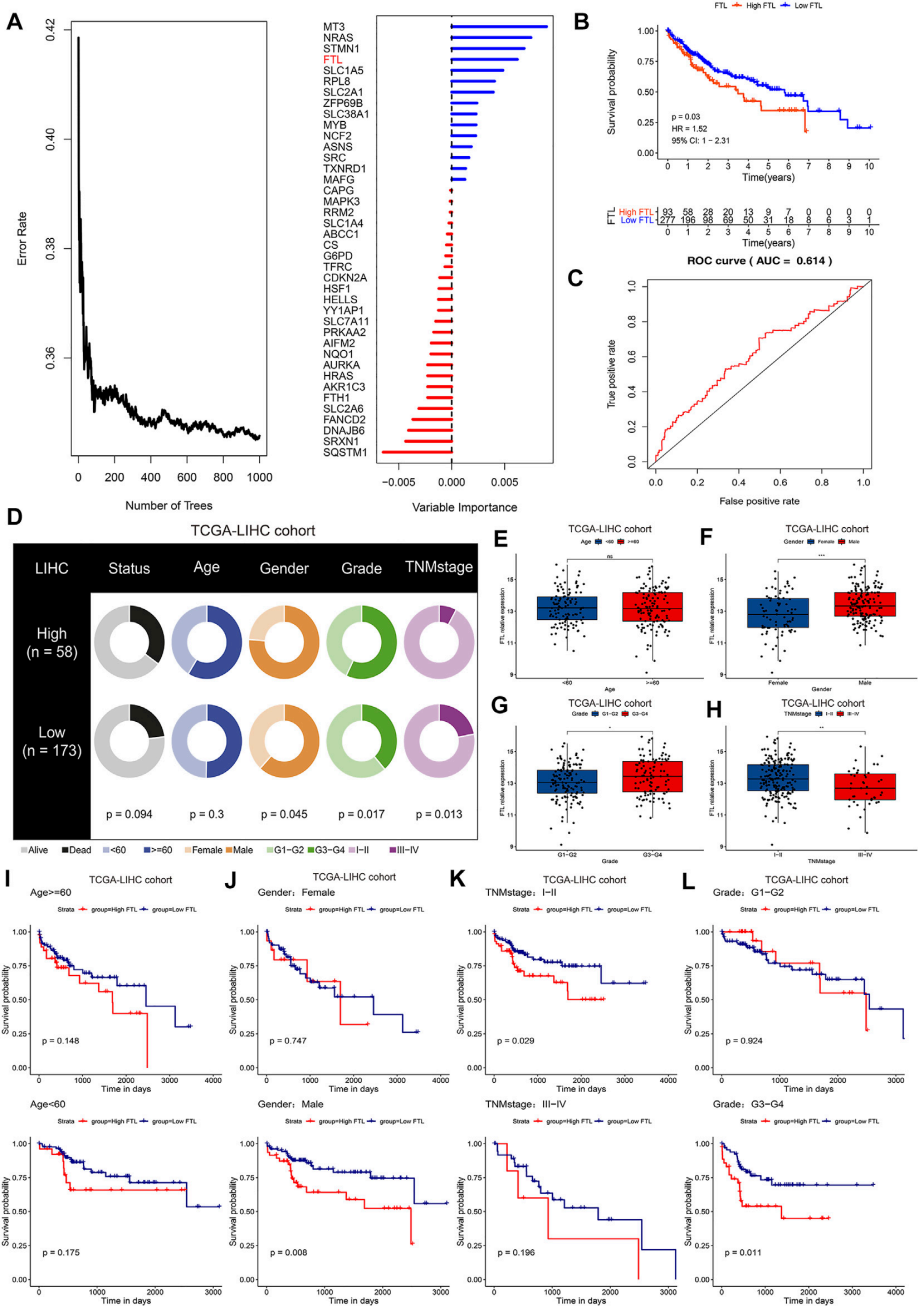
FTL is a critical ferroptosis regulator associated with prognosis and clinical features in HCC. **(A)** Random forest analysis indicating the importance of ferroptosis regulators in predicting overall survival in HCC. **(B)** K-M survival curve showing the different overall survival rates in the high FTL and low FTL groups in the TCGA-LIHC cohort. **(C)** ROC curves evaluating the specificity and sensitivity of FTL in predicting overall survival in HCC. **(D)** Differences in clinical features, including survival status, age, sex, grade, and TNM stage, between the high FTL and low FTL groups in the TCGA-LIHC cohort. **(E–H)** Correlation of FTL expression and clinical features including age, sex, grade, and TNM stage in the TCGA-LIHC cohort. **(I-L)** K-M survival curve showing the different overall survival rates of patients in the high FTL and low FTL groups in different clinical subgroups of the TCGA-LIHC cohort. ns, no significance. *p < 0.05, **p < 0.01, and ***p < 0.001.

Next, random forest analysis was used to analyze critical ferroptosis-related genes in HCC, and the results indicated that MT3, NRAS, STMN1, FTL, and SLC1A5 were the five most important ferroptosis regulators in HCC associated with prognosis (Figure 3A). According to previous publications, there have been a lot of research on the function of three genes NRAS (Dietrich et al., 2019; Ding et al., 2020), STMN1 (Zhang et al., 2020), and SLC1A5 (Zhao et al., 2021) in HCC, and in this study, our pre-experiment indicated the expression of MT3 didn’t affect the proliferation of HCC, so we focused on the role of FTL in HCC. With the FTL expression level at 75% by quartile as the criterion, we divided the high FTL group and the low FTL group in the TCGA-LIHC cohort. The K-M survival curve and ROC curve showed that higher FTL expression predicted poor survival with superior reliability in HCC patients (Figures 3B,C). Figures 3D–H indicates that the expression level of FTL is closely associated with clinical features (*p* < 0.05), including sex, history grade, and TNM stage, but not age, in HCC. Interestingly, the predictive value of FTL was also closely related to clinical features, including sex, history grade, and TNM stage (*p* < 0.05), but not age in HCC. High expression levels of FTL showed poor survival time in the HCC patients with male sex, TNM stage I-II, and G3-G4 grade (*p* < 0.05), however, in HCC patients with female sex, TNM stage III-IV, and G1-G2 history grade, there was no sign of FTL expression in the prognosis of HCC (Figure 3I-L).

### Measuring FTL Expression and Validating the Diagnostic Ability of FTL in HCC Patients

**FIGURE 4:**
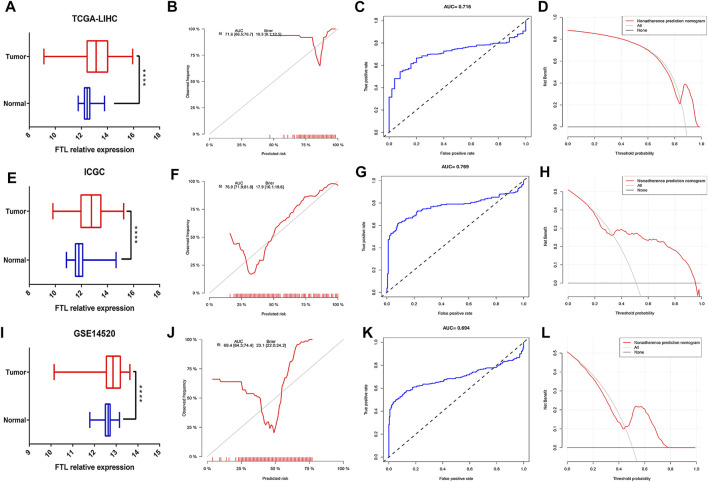
Measurement of FTL expression in tumor samples compared with normal samples and evaluation of the diagnostic ability of FTL in HCC. **(A,E,I)** Box plot showing the FTL mRNA expression level in normal and tumor tissues in HCC from the TCGA-LIHC cohort, ICGC-HCC cohort, and GSE14520 cohort. **(B,F,J)** Calibration curves indicating the diagnostic ability of FTL in HCC patients from the TCGA-LIHC cohort, ICGC-HCC cohort, and GSE14520 cohort. **(C,G,K)** ROC curves assessing the predictive performance of the diagnostic model in the TCGA-LIHC cohort, ICGC-HCC cohort, and GSE14520 cohort. **(D,H,L)** DCA curves validating the predictive potency of the diagnostic model in the TCGA-LIHC cohort, ICGC-HCC cohort, and GSE14520 cohort. ****p < 0.0001.

To verify the expression level of FTL in normal tissue and tumor tissue in HCC patients, we obtained three different HCC cohorts, including the TCGA-LIHC cohort, ICGC HCC cohort, and GSE14520 cohort. The results indicated that FTL was obviously higher in HCC tissue than in normal tissue (Figures 4A,E,I). Then, calibration curves, ROC curves, and decision curve analysis (DCA) were used to validate the diagnostic ability of FTL in HCC patients. The AUC values of the calibration curve and ROC curve were 0.716, 0.769, and 0.694, respectively, in these three cohorts (Figures 4B,C, F–G, J–K), indicating good predictive performance of this diagnostic model. The DCA curve of our diagnostic model showed some net benefit for prediction (Figures 4D,H,L). Overall, these results indicated that FTL was more highly expressed in HCC tissue and that the diagnostic model showed good predictive performance for distinguishing between HCC samples and normal samples.

### Identification of the Different Immune Microenvironment Between High FTL and Low FTL HCC Patients

**FIGURE 5:**
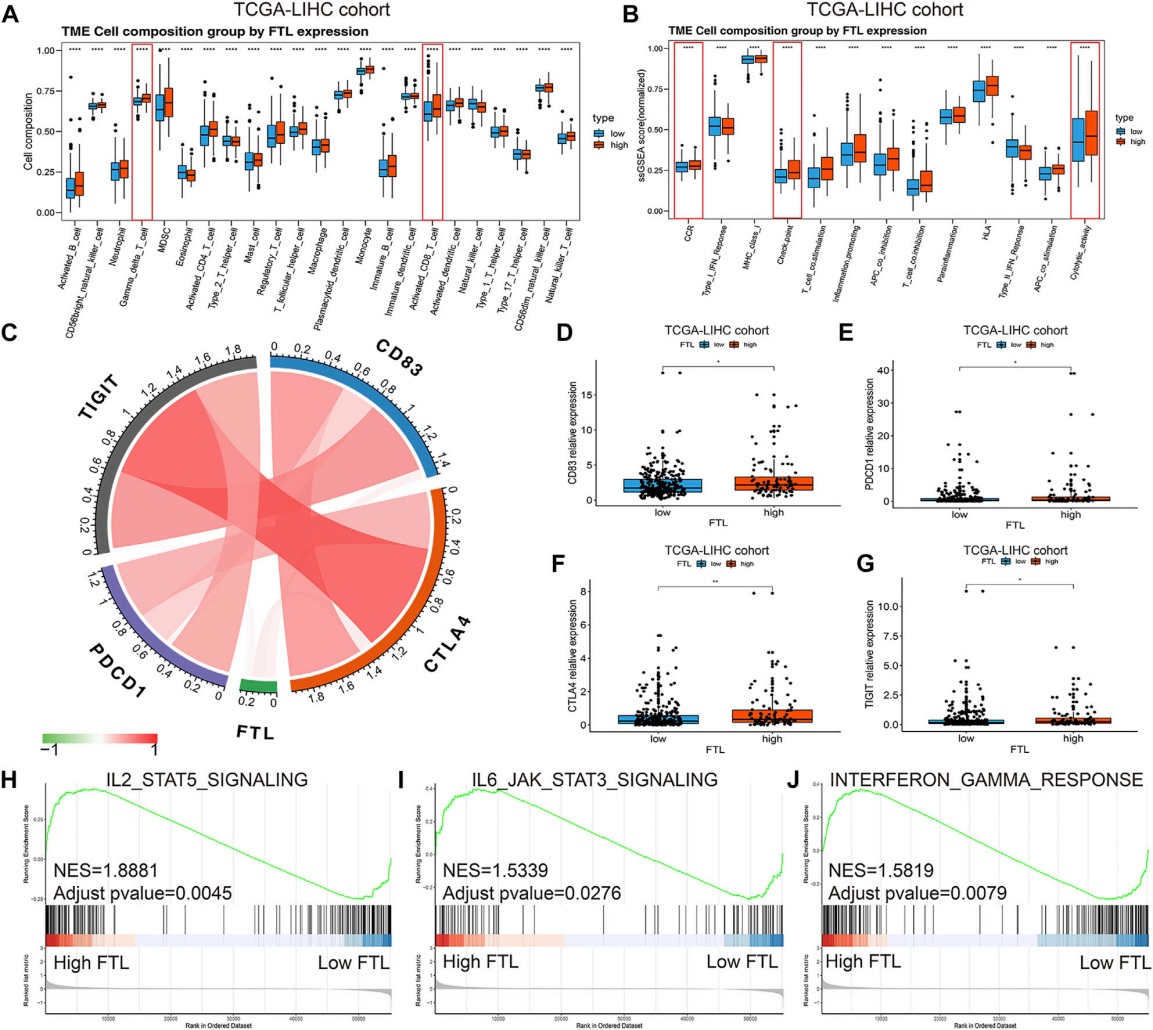
Identification of immune cell infiltration and immune activity between high FTL and low FTL HCC patients. **(A)** Box plot showing immune cell infiltration in HCC patients in the high FTL group compared with the low FTL group. **(B)** Box plot showing the different immune activities between high FTL and low FTL HCC patients. **(C)** Relationship of FTL expression and immune checkpoints, including PDCD1, CTLA4, TIGIT, and CD83, in HCC. **(D–G)** Expression level of checkpoint genes, including PDCD1, CTLA4, TIGIT, and CD83, between high FTL and low FTL HCC patients. **(H–J)** GSEA shows the correlation between FTL expression and immune activation-related signaling pathways, such as the IL2-STAT5 signaling pathway, IL6-JAK-STAT3 signaling pathway, and interferon-gamma response signaling pathway. *p < 0.05, **p < 0.01, and ****p < 0.0001.

To further clarify the immune microenvironment associated with the FTL expression level in HCC, a single sample gene set enrichment analysis (ssGSEA) was used to identify immune cell infiltration and immune activity. The results showed that higher expression indicated more immune cell infiltration, including activated CD8^+^ T cells and Gamma delta T cells (Figure 5A). In addition, the enrichment scores of cytolytic activity, CCR, HLA, and immune checkpoint were significantly increased in high FTL HCC patients (Figure 5B). Figure 5C shows the relationship between FTL expression and immune checkpoints, including PDCD1, CTLA4, TIGIT, and CD83; these checkpoints were expressed at higher levels in the high FTL group than in the low FTL group (Figures 5D–G). GSEA indicated that high FTL expression was also positively associated with immune activation-related signaling pathways, including the IL2-STAT5 signaling pathway, IL6-JAK-STAT3 signaling pathway, and interferon-gamma response signaling pathway (Figures 5H–J). This evidence suggests that FTL expression is positively associated with the immune-activation microenvironment in HCC.

### Prediction of Chemotherapeutic and Targeted Therapeutic Responses Between High FTL and Low FTL HCC Patients

**FIGURE 6:**
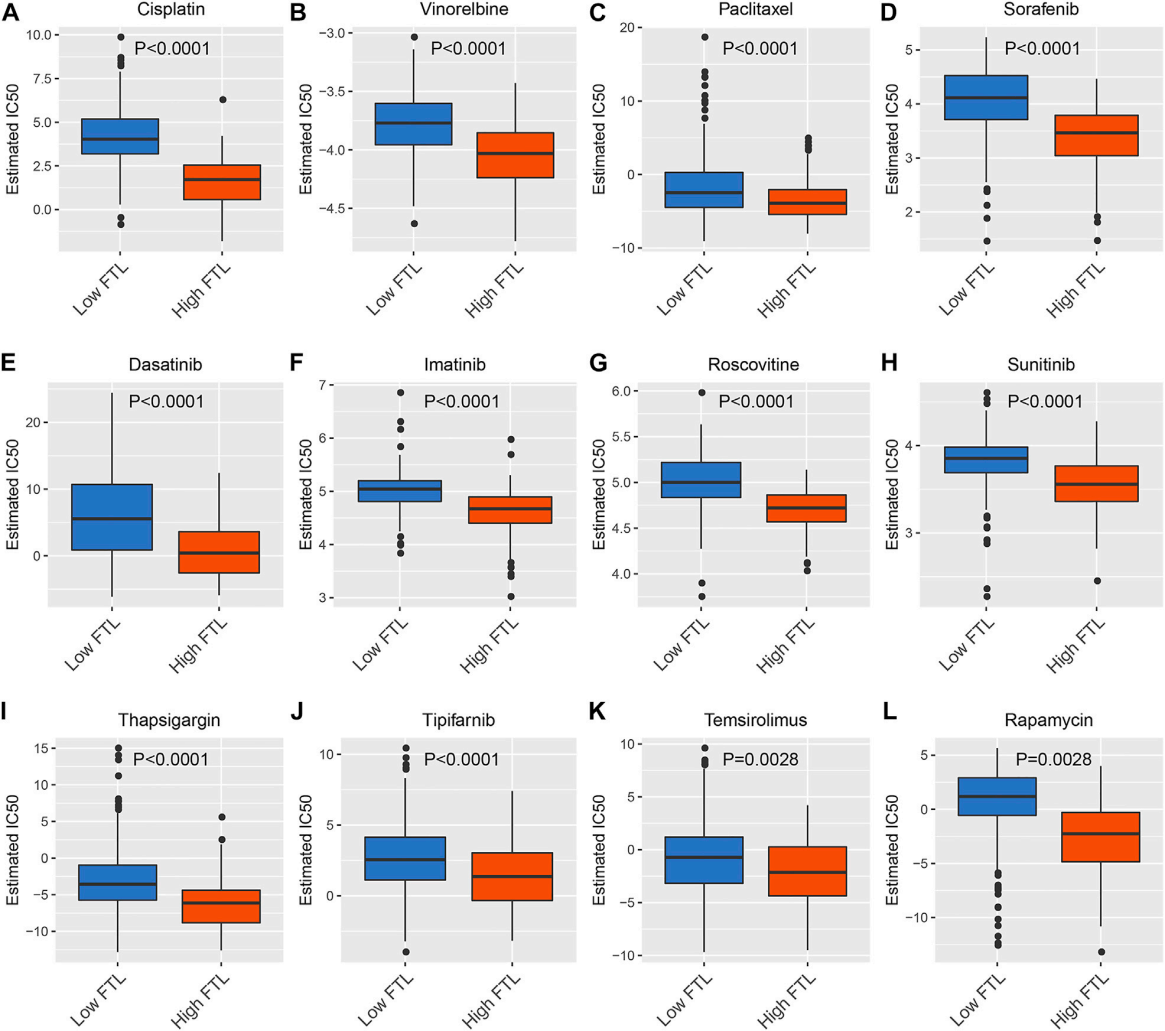
Prediction of chemotherapeutic and targeted therapeutic responses in HCC patients with high FTL and low FTL. **(A–C)** Box plot showing the prediction of the IC50 value of traditional chemotherapeutic drugs, including cisplatin, paclitaxel, and vinorelbine, between the high FTL and low FTL groups of HCC patients. **(D-L)** Box plot showing the prediction of the IC50 value of targeted drugs, including sorafenib, dasatinib, imatinib, roscovitine, sunitinib, thapsigargin, tipifarnib, temsirolimus, and rapamycin, in HCC patients with high FTL compared with the low FTL group.

To predict the chemotherapeutic and targeted therapeutic responses, half the maximum inhibitory concentration (IC50) of 266 anticancer drugs was obtained from the Genomics of Drug Sensitivity in Cancer (GDSC) website. The results showed that traditional chemotherapeutic drugs, including cisplatin, paclitaxel, and vinorelbine, had lower IC50 values in the high FTL group than in the low FTL group in HCC (*p* < 0.05) (Figures 6A–C). In addition, HCC patients with higher FTL expression showed lower IC50 values of targeted drugs such as sorafenib, dasatinib, imatinib, roscovitine, sunitinib, thapsigargin, tipifarnib, temsirolimus, and rapamycin (*p* < 0.05) (Figure 6D–L). These results indicate that the expression level of FTL is related to the sensitivity of some chemotherapeutic drugs and targeted drug treatments.

### Construction and Validation of a Nomogram Integrating Independent Predictive Factors

**FIGURE 7:**
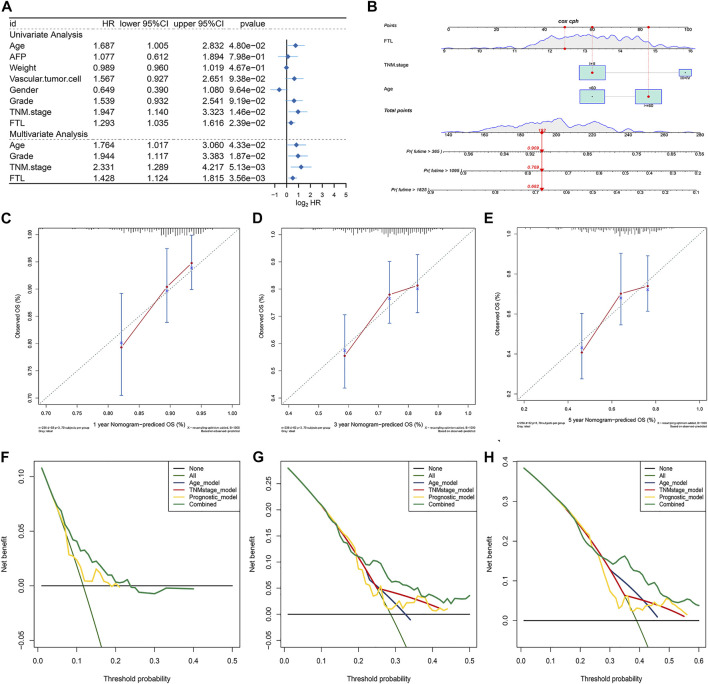
Construction and validation of a nomogram integrating independent predictive factors. **(A)** Univariate and multivariate Cox regression for evaluating the independent prognostic factor from FTL and other clinical features, such as age, AFP, weight, vascular invasion, sex, history grade, and TNM stage. **(B)** Nomogram constructed for predicting the survival probability of patients with HCC at 1, 3, and 5 years. **(C–E)** Calibration curve of the nomogram for the overall survival probability of HCC patients at 1, 3, and 5 years. **(F–H)** DCA curve was used to evaluate the predictive ability of single independent predictive factors compared with the nomogram in HCC patients at 1, 3, and 5 years.

To evaluate the independent performance of the FTL level in predicting prognosis compared with other clinical features, including age, AFP, weight, vascular invasion, sex, history grade, and TNM stage, univariate and multivariate Cox regression were used. The results showed that age, TNM stage, and FTL level were independent predictive factors in HCC patients (Figure 7A). Then, a nomogram predictive model was constructed based on these three independent predictive factors to quantify the survival probability of HCC patients at 1, 3, and 5 years (Figure 7B). The calibration curve of the nomogram for predicting the overall survival probability of HCC patients at 1, 3, and 5 years was close to the 45° line (Figures 7C–E). DCA was used to evaluate the guiding significance of these independent predictive factors for predicting overall survival time at 1, 3, and 5 years, and the results show that the nomogram has superior predictive value in clinical practice (Figures 7F–H). These results showed that FTL is an independent prognostic factor and that our nomogram has valuable predictive performance.

### Silencing FTL Expression Inhibited HCC Cell Proliferation and Triggered Ferroptosis

**FIGURE 8:**
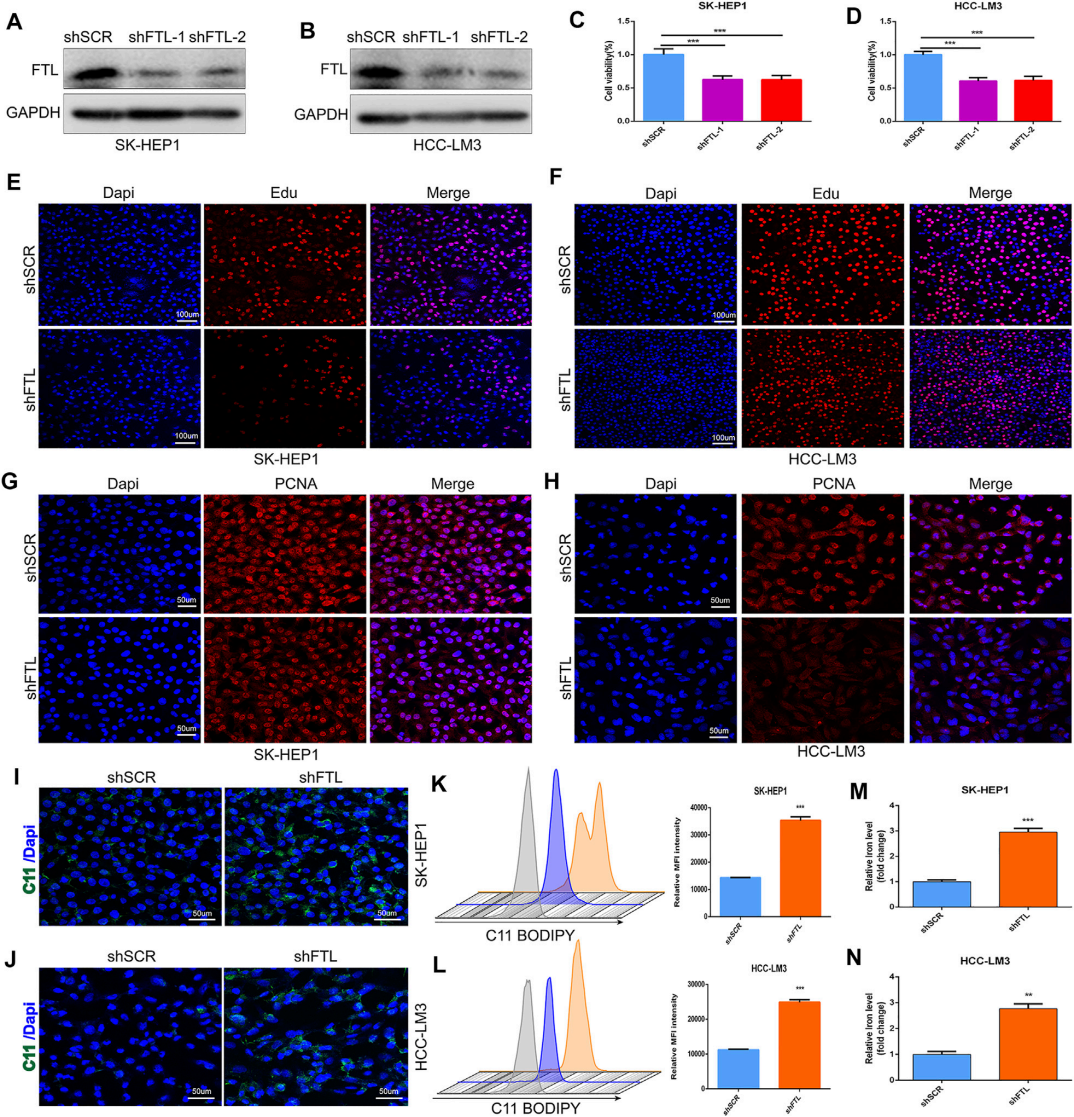
FTL knockdown inhibited HCC-cell proliferation and induced ferroptosis in HCC cells. **(A, B)** Western blot indicated the FTL inhibition efficiency of FTL shRNA administration in SK-HEP1 and HCC-LM3 cells. **(C–F)** The proliferation of SK-HEP1 and HCC-LM3 cells was measured by using CCK8 kit, Edu assay. **(G–H)** The protein level of PCNA was measured by immunofluorescence assay. **(I–L)** The lipid peroxidation level of SK-HEP1 and HCC-LM3 cells was detected by using C11 BODIPY probe. **(M–N)** The concentration of iron level in SK-HEP1 and HCC-LM3 cells with or without shRNA administration. Data are shown as the mean ± SD of at least three independent experiments. **p < 0.01 and ***p < 0.001.

To confirm the role of FTL in the tumor progression of HCC, HCC cells including SK-HEP1 and HCC-LM3 were transfected with two different FTL shRNA and scramble shRNA. Western blot indicated the FTL shRNA effectively inhibited FTL expression in SK-HEP1 and HCC-LM3 cells (Figures 8A,B). Then CCK8 kit and Edu assay were used to assess the role of FTL on HCC-cell proliferation. The result showed FTL inhibition obviously suppressed the proliferation of SK-HEP1 and HCC-LM3 cells (Figures 8C–F). To further explore the role of FTL in HCC-cell proliferation, immunofluorescence was used to measure the expression of PCNA in SK-HEP1 and HCC-LM3 cells. The result indicated FTL inhibition effectively decreased PCNA expression levels in HCC cells (Figures 8G,H). Moreover, silencing FTL expression induced lipid peroxidation levels of SK-HEP1 and HCC-LM3 cells (Figure 8I-L) and the concentration of iron inSK-HEP1 and HCC-LM3 cells was significantly increased with FTL inhibition (Figure 8M–N). These results indicated FTL inhibition effectively suppressed HCC-cell proliferation and triggered ferroptosis in HCC cells.

## Discussion

Hepatocellular carcinoma is the most common type of liver cancer and the second leading cause of cancer-related death (Ferlay et al., 2015). Early HCC usually has no obvious clinical manifestations, and most of the symptoms are already advanced with a poor prognosis. Current treatment options include surgical resection, liver transplantation, chemotherapy, and targeted drug therapy for advanced HCC. However, surgical treatment is only applicable to patients with early HCC with very strict indications, and HCC patients are often diagnosed as advanced and have drug resistance to chemotherapy and targeted drugs (Llovet et al., 2021). Therefore, it is very important to find targets related to the diagnosis and prognosis of HCC.

Ferroptosis, first named in 2012 (Hirschhorn and Stockwell, 2019), is a nonapoptotic form of programmed cell death driven by iron-dependent phospholipid peroxidation (Jiang et al., 2021). Iron enrichment in the microenvironment of malignant tumors further promotes the malignancy of tumors, and the liver is an organ prone to oxidative damage (Toyokuni, 2009). Therefore, the study of ferroptosis in HCC is conducive to finding new therapeutic targets for HCC. Sorafenib is the main targeted drug for the treatment of advanced HCC, but its resistance limits its efficacy. At present, it is considered that enhancing the ferroptosis sensitivity of HCC is an effective way to solve sorafenib resistance, such as upregulation of MT-1G or antagonism of NRF2 (Sun et al., 2016a; Sun et al., 2016b). A series of prognostic models based on the relationship between ferroptosis and HCC is helpful to accurately grasp the condition of HCC. For example, the iron death-related gene model can predict the survival rate of HCC patients, the prognostic model of HCC ferroptosis-related regulators can predict the prognosis of HCC patients and the choice of treatment methods more accurately, and the iron death-related prognostic model constructed by combining the methylation characteristics of some HCC can predict the risk more accurately (Du and Zhang, 2020; Liang et al., 2020; Deng et al., 2021; Xu et al., 2021). In this study, we comprehensively analyzed the potential mechanism and prognostic role of 239 ferroptosis-related genes in HCC. Then, these prognostic ferroptosis regulators were divided into markers, suppressors, and drivers of ferroptosis. Interestingly, based on these prognostic ferroptosis-related genes, HCC was separated into two different subtypes, fesclusters A and B. The overall survival of fescluster B was obviously poor than that of fescluster A. In addition, the fescluster was closely related to sex, age, vascular invasion, histological grade, and survival status in HCC. More importantly, we found that FTL was a critical ferroptosis regulator in HCC. A high expression level of FTL predicted a worse survival rate, and FTL functioned as an independent prognostic and diagnostic factor in HCC.

The tumor microenvironment (TME) is the cellular environment influencing the process of tumors and is composed of immune cells (both innate and acquired immune cells), nonimmune stromal cells (such as fibroblasts, endothelial cells, and various tissue-associated cells), and extracellular matrix proteins (Monteran and Erez, 2019; Wculek et al., 2020; Pansy et al., 2021). Among them, all kinds of cells interact with each other and cancer cells through the secretion of various cytokines, chemokines, and other signals, so they play a key role in the regulation of the tumor immune response. The TME plays an important role in the regulation of HCC (Leonardi et al., 2012; Wu et al., 2019). For example, hepatic stellate cells (HSCs) promote tumorigenicity by eliminating HCC necrosis, and some secreted cytokines and chemokines are essential for the stem-like characteristics of HCC cells (Amann et al., 2009; Xiong et al., 2018). While immune cells can inhibit the development of HCC, immune cell defects lead to immunosuppression of HCC (Piñeiro Fernández et al., 2019; Chen et al., 2021). In this study, we found that a high FTL expression level indicated increased infiltration of immune cells, such as activated CD8^+^ T cells and Gamma delta T cell. High FTL expression was also associated with immune-related signaling pathways, including the IL2-STAT5 signaling pathway and the interferon-gamma response signaling pathway. In addition, the enrichment scores of cytolytic activity, CCR HLA, and immune checkpoint were significantly increased in high FTL HCC patients. Moreover, immune checkpoints, including PDCD1, CTLA4, TIGIT, and CD83, were positively associated with FTL expression levels in HCC. This evidence suggests that FTL may function as a predictor of the immune response and that patients with high FTL levels may obtain more clinical benefits from immunotherapy in HCC.

FTL is a ferritin light chain that forms ferritin with FTH to complete the storage function of iron. Due to its structural stability, FTL mainly exists in organs rich in iron storage and directly affects iron homeostasis (Li et al., 2015). Current studies on the function of FTL in HCC mainly focus on its role in iron death. It has been reported that FTL plays a role in the process of apoptosis (Fan et al., 2009), but more studies regard FTL as a factor related to iron metabolism and consider it to play a role in ferroptosis. Gsk-3β affects ferroptosis by antagonizing iron metabolites, including FTL, and disrupting iron homeostasis (Wang et al., 2021a). Transcriptional inhibition of FTL has also been reported to increase the sensitivity of cancer cells to ferroptosis in lung adenocarcinoma (Wang et al., 2021b). In this study, we found that the level of FTL expression was also associated with immunotherapy and molecular targeted therapy, including sorafenib and imatinib. In addition, FTL was highly expressed in HCC tumor tissue and served as a promising prognostic and diagnostic factor in HCC patients. Moreover, silencing FTL expression effectively suppressed HCC-cell proliferation and inhibited PCNA expression in HCC cells. FTL inhibition increased the levels of lipid peroxidation and ferric ion levels in HCC cells. This evidence indicates that FTL can be considered a critical ferroptosis regulator and a novel therapeutic target for HCC.

However, there are some limitations to our research. All RNA sequence data and clinical information in this study were obtained from public databases, such as the TCGA database, GEO database, and ICGC database. The predictive role of FTL in the response to immunotherapy and molecular targeted therapy in HCC patients needs to be validated in a clinical cohort, and a public database lacks a feasible cohort. More experiments, such as animal models and molecular biology experiments, are needed to explore the oncogenic effect of FTL and the potential regulatory mechanism in HCC.

## Data Availability

The original contributions presented in the study are included in the article/Supplementary Material, further inquiries can be directed to the corresponding author.
