# Supplementary material for: Integrated Analysis Reveals Critical Ferroptosis Regulators and FTL Contribute to Cancer Progression in Hepatocellular Carcinoma
Source: Front Genet. 2022 May 16;13:897683. doi: 10.3389/fgene.2022.897683 (PMC9149379; doi:10.3389/fgene.2022.897683)
Supplement: Supplementary file 2 [file Table1.DOCX]

**Details of the used antibodies**

| Name | Dilution | Species | Manufacture | Cat, No. | Application |
| --- | --- | --- | --- | --- | --- |
| Anti-Ferritin Light Chain | 1:1000 | Rabbit | Abcam | ab69090 | Western blot |
| Anti-GAPDH | 1:4000 | Mouse | Abcam | ab8245 | Western blot |
| Goat anti Rabbit IgG-HRP | 1:10000 | Goat | ABclonal | AS014 | Western blot |
| Goat anti Mouse IgG-HRP | 1:10000 | Goat | ABclonal | AS003 | Western blot |
| PCNA | 1:400 | Rabbit | [Cell Signaling Technology](https://www.baidu.com/link?url=ne_k8s24UxhaxCv9Y_LP58qZzV41MHKhKZy9Ul9MGxKZIu2VwbSZ79Ryp1xAd6jS&wd=&eqid=e96abed800009992000000046043551d) | #13110 | IF |
